# Betacyanins and Anthocyanins in Pulp and Peel of Red Pitaya (*Hylocereus polyrhizus* cv. Jindu), Inhibition of Oxidative Stress, Lipid Reducing, and Cytotoxic Effects

**DOI:** 10.3389/fnut.2022.894438

**Published:** 2022-06-23

**Authors:** Hock Eng Khoo, Xuemei He, Yayuan Tang, Zhichun Li, Changbao Li, Yuan Zeng, Jie Tang, Jian Sun

**Affiliations:** ^1^Agro-Food Science and Technology Research Institute, Guangxi Academy of Agricultural Sciences, Nanning, China; ^2^College of Chemistry and Bioengineering, Guilin University of Technology, Guilin, China; ^3^Guangxi Key Laboratory of Fruits and Vegetables Storage-Processing Technology, Nanning, China; ^4^Division of International Cooperation, Guangxi Academy of Agricultural Sciences, Nanning, China

**Keywords:** antioxidant activity, betacyanins, cardioprotective effect, dragon fruit, free radical

## Abstract

This study aimed to promote red pitaya fruit parts as alternate sources of nutraceuticals. The red pitaya of Chinese origin was determined for its *in vitro* efficacy, where the fruit extracts were evaluated based on the selected antioxidative properties, lipid-reducing capacity, and cytotoxicity. The betanin, total betacyanins, total anthocyanins, and DPPH radical scavenging activity of the red pitaya pulp and peel extracts were determined by spectrophotometric analyses. Cell culture assays were used to examine *in vitro* efficacy and cytotoxicity of the pitaya extracts. The result showed that red pitaya peel extract had a higher total betacyanins and total anthocyanins content than the pulp extract, but the peel extract had a lower DPPH radical scavenging effect than the pulp extract. The red pitaya extracts also had a protective effect in reducing oxidative stress, especially the peel extract. All fruit samples had a low anticancer potential except for betanin and anthocyanin standards. The protective effect of pitaya peel could be attributed to betacyanins and anthocyanins. Both pulp and peel extracts had a weak anticancer effect because these extracts contained polysaccharides and other phytochemicals that were not cytotoxic. As the peel extract of red pitaya was not cytotoxic, it is a potent source of betacyanins for reducing oxidative stress.

## Introduction

Tropical fruits are rich in biologically active ingredients such as alkaloids, anthraquinones, phenolics, and terpenoids. These compounds can scavenge free radicals (1, 2). Among the tropical fruits, pitaya is the fruit under the cactus family (Cactaceae). Pitaya belongs to the genus *Hylocereus*; it is commonly called dragon fruit because it looks like a dragon ball. Three species of pitaya are widely cultivated in Indochina and China. They are white, red, and yellow pitayas. The scientific names of these three pitaya species are *Hylocereus undatus*, *H. polyrhizus*, and *H. megalanthus*, respectively.

A few varieties of red pitaya are widely cultivated in China. They are Hainan Mibao, Jindu No. 1, Tainong No. 1, and Vietnam H14. Nutritional compositions and antioxidant properties of red pitaya have been reported previously (3, 4). Red pitaya is one of the tropical fruits that contain a high amount of betalains. Betacyanins and betaxanthins are betalains found in fruits and vegetables. Betacyanins are red pigments found in red pitaya, whereas betaxanthins are yellow pigments determined in yellow pitaya. Betaxanthin in red pitaya pulp has never been studied. Betacyanins in red pitaya comprise betanin as the main bioactive component (5). Other betacyanins identified in the red pitaya are isobetanin, bougainvillein-r-I, phyllocactin, and iso-phyllocactin (6).

Betacyanins, the red-colored pigments found in the red pitaya pulp, are unstable at higher pHs and temperatures. A previous study measured the absorbances of the red pitaya extract solutions at 537 nm. The extracts were treated with different pHs (3, 5, and 7) and temperatures (4 and 25°C). The result showed that the extract solutions treated with a pH higher than 7.0 and a temperature of 25°C had significantly lower absorbances than the other treatment conditions (7). The finding revealed that solvent pH of >7 and extraction temperature of 25°C or higher caused rapid degradation of betacyanins in the red pitaya extract.

Betanin is known for its high free radical scavenging activity and inhibition of oxidative stress (4, 8). The compound has a higher antioxidant activity than ascorbic acid (9). The antioxidant capacities of betanin have been tested using several antioxidant approaches, such as scavenging of 2,2-diphenyl-1-picrylhydrazyl (DPPH), galvinoxyl, superoxide, and hydroxyl radicals (8). The antioxidative effects were dose-dependent. The study also reported the cytotoxic effect of betanin on three types of cancerous cells (HT29, Huh7, and PON1-Huh7). The antioxidant effect could be attributed to hydroxyl and imino groups of betanin (10). Moreover, beetroot betanin has been reported as one of the anticancer agents for inhibiting the proliferation of several types of cancer cell lines (11). The inhibition of adipogenesis in adipocytes by pure betanin has also been reported in the literature (12).

Anthocyanins have been determined in red pitaya besides betacyanins. Literature showed that several anthocyanins had been identified in the pulp and peel of red pitaya and the peel of white pitaya. These anthocyanins were cyanidin 3,5-O-diglucoside, cyanidin 3-O-diglucoside-5-O-glucoside, delphinidin 3-O-glucoside, 4’-O-methyldelphinidin 3-O-glucoside, peonidin 3-O-diglucoside-5-O-glucoside, peonidin 3-O-sambubioside-5-O-glucoside, isopeonidin 3-O-arabinoside, and petunidin 3-O-(6”-acetyl-glucoside) (13). Another study reported that cyanidin (31.63%), delphinidin (7.53%), and malvidin (7.37%) had been identified in red pitaya peel extract and its fractions (14). The fresh pulp and peel of red pitaya also had total anthocyanins content of 159.7 ± 8.9 and 135.4 ± 9.3 mg/g dried sample, respectively (15). Although anthocyanins are not the main phytochemicals determined in the red pitaya samples, they are polyphenolic bioactives with several health benefits. The possible health benefits of anthocyanins are anticancer, antidiabetes, antimicrobial, antiobesity, cardioprotection, neuroprotection, and improved visual functions (16).

The methanolic extracts of red pitaya of Chinese origin have not been widely studied. The lipid-reducing effect of betacyanin-rich extracts of the red pitaya pulp and peel has not yet been determined. The existing studies have reported possible cardioprotective and anticancer effects of red pitaya pulp but not for the fruit peel. Therefore, this study aimed to promote methanolic extracts of red pitaya pulp and peel and their biologically active substances, such as betacyanins and anthocyanins, as nutraceuticals. We hypothesized that the protective effect of betacyanin-rich red pitaya peel against oxidative stress was better than that of the fruit pulp. The findings could also provide a theoretical basis for understanding the antioxidative properties of red pitaya pulp and peel in maintaining good health.

## Materials and Methods

### Sampling and Sample Preparation

A 5-kg red pitaya (*H. polyrhizus* cv. Jindu) was purchased from a wholesaler market in the Nanning city of China. The fruit was washed and air-dried before separating the peel. The fruit samples were cut into smaller pieces before freeze-drying. All fruit samples were freeze-dried using a Pilot10-15M vacuum freeze dryer (Biocool, Beijing, China). The lyophilized samples were pulverized, passed through a 20-mesh sieve, and kept in a freezer (Haier Group, China) at –20 °C before extraction. The powder form of commercially available red pitaya was purchased from a local supplier. It has 200-mesh granules. The commercial type of colorant produced from red pitaya was named colorant. It was used as a control sample.

### Extraction of Sample for Antioxidative Analyses

The extraction of antioxidants in the pitaya sample was performed by dissolving a 2.0 g sample with 20 mL of 80% methanol in a 250 mL Erlenmeyer flask. The extraction method was adapted from Albano et al. (17). The flask that had the extraction mixture was rotated for 1 h at room temperature (RT) of 25°C, and the residue was separated using a 125 μm nylon mesh fabric. The filtrate was centrifuged at 3,000 rpm for 10 min at RT, and the residue was re-extracted with the same volume of the extraction solvent another five times. The supernatants were collected as extract solution, and it was subjected to rotary evaporation at 49°C to remove the organic solvent before freeze-drying. All lyophilized extracts were stored at –20°C before further analyses. Triplicate extractions were performed to obtain highly repeatable data.

### Estimation of Total Betacyanins and Betanin Content

The betanin and total betacyanins content (TBC) of the pulp and peel extracts of red pitaya were estimated using a microplate spectrophotometer by referring to the method described by Phebe et al. (5) with some modifications. In brief, 100 μL of the extract at an extract concentration of 100 μg/mL was prepared and then pipetted into the wells of a 96-well plate. The absorbance was measured using an Epoch microplate spectrophotometer (BioTek Instruments, Inc., Winooski, VT, United States) at 538 nm. The TBC content was calculated based on the standard linear calibration curve (y = 0.0078x - 0.0296, R^2^ = 0.9922) of the pure betanin at concentrations ranging from 10 to 80 μg/mL. The betanin content of the red pitaya extracts was calculated using the Equation 1 reported in the literature (18, 19).


(1) $$\mathrm{Betanin}\,\mathrm{content}\,\left(\mathrm{mg}/g\,\mathrm{extract}\right)=\frac{\text{A}\times \text{MW}\times \text{V}\times \text{DF}\times 1000}{\text{E}\times \text{L}\times \text{W}}$$

Where A is the absorbance at 538 nm, MW is the molecular weight of betanin (550 g/mol), V is the volume of extract, DF is the dilution factor, E is the molar extinction coefficient of betanin (60,000 M^–1^ cm^–1^), L is the path length (0.286 cm), and W is the weight of the extract.

### Estimation of Total Anthocyanins Content

The total anthocyanins content (TAC) of the red pitaya extracts was estimated based on the method described by Khoo et al. (20) with a slight modification. The absorbance of the diluted fruit extract solution (2 mL) was measured at 538 nm instead of at 535 nm due to the absorption maximum of the extract solution was 538 nm. TAC was calculated based on the equation as follows:


(2) $$\mathrm{TAC}\,\left(\mathrm{mg}/g\right)=\frac{\text{A}\times \text{V}\times \text{N}}{98.2\times \text{L}\times \text{m}}$$

Where TAC is the total anthocyanins content of the fruit extract, A is the absorption value measured at 538 nm, V is the volume of the extract solution (mL), N is the dilution factor, 98.2 is the extinction coefficient (M^–1^ cm^–1^) at 538 nm, L is the path length (1 cm), and m is the mass of the fruit extract.

### Estimation of Sugar Content

The total sugar content of the red pitaya extracts was estimated using a 3810 (PAL-1) digital pocket refractometer (Atago Co., Ltd, Tokyo, Japan) at RT of 25 ± 1°C. In brief, 1 mL of the extract was pipetted onto the prism of the sample stage of the refractometer (21). The total sugar content was recorded as Brix value (%) in triplicate.

### DPPH Radical Scavenging Activity

DPPH radical scavenging activity of the red pitaya extracts was performed by referring to the method described by Kong et al. (22) with slight modification. Briefly, 20 μL of different concentrations of extracts (0.015625-10 μg/mL) and standards (0.03125-80 μg/mL) were added to 96-well plates and then added with the DPPH (100 μL, 0.5 mM in methanol) and Tris–HCl (80 μL, 0.1 M) solutions. The antioxidant standards consisted of betanin and cyanidin-3-glucoside (C3G). The mixture was then homogenized and left to stand at RT for 30 min. The microplate spectrophotometer was used to obtain the absorbance of the DPPH-mixture by measuring at 517 nm. The absorbances were measured in triplicate; the percentage of antioxidant activity (AA) of the DPPH assay was calculated according to equation as follows:


(3) $$\mathrm{AA}\left(\%\right)=\frac{\left(\mathrm{A0}-\mathrm{At}\right)}{\mathrm{A0}}\times 100$$

Where A0 is the absorbance of the control, and At is the absorbance of the sample. EC_50_ values of the fruit (extracts), also known as effective concentrations for 50% inhibition, were calculated based on the equations obtained from the linear calibration curves, where the R^2^ values of the equations were higher than 0.9. A lower EC_50_ value indicates a higher antioxidant activity.

### Cell Culture Assays

Human hepatocellular carcinoma (HepG2, ATCC HB-8065), rat pancreatic (RIN-5F, ATCC-2057), and mouse embryonic fibroblast (3T3-L1, ATCC CL-173) cells within 2-5 passages were used to determine the anticancer, lipid-reducing effect, and cytotoxicity of different extract concentrations of the red pitaya extracts (78.13–10 mg/mL) and pure standards (1.25-80 μg/mL). The cell culture assays were performed to evaluate the inhibition of oxidative stress, lipid reducing effect, cell cytotoxicity, and anticancer potential according to the method described in the literature (23). In brief, the cells (100 μL, 1 × 10^5^/well) were fixed and cultured in a 96-well culture plate.

HepG2 cells were cultured in a complete medium containing high-glucose DMEM (4.5 g/L D-glucose), fetal bovine serum (FBS), and penicillin-streptomycin solution (Gibco, China). The complete medium used to culture 3T3-L1 cells contained 96% DMEM (1.0 g/L D-glucose), 10% newborn calf serum, 1% antibiotic solution, and 3% growth supplements. The growth supplements purchased from Invitrogen (China) consisted of GlutaMAX, non-essential amino acids (100×), and 100 mM sodium pyruvate (1:1:1). RIN-5F cells were cultured in a complete medium containing RPMI 1640 medium, FBS, and the antibiotic solution (Gibco, China). The culture solutions were prepared according to the ratio of 89:10:1, except for the 3T3-L1 cells. A cooled 0.25% (w/v) trypsin-EDTA solution was used to detach the cells from the culture flasks.

All cells were grown to 70% confluence in the 96-well plate and washed with phosphate buffer saline (PBS, Solarbio Life Sciences, Beijing, China) twice before adding 0.1 mL extract solution or pure standard and 0.1 mL of the complete medium. The red pitaya extract or pure standard was dissolved in the culture medium containing 0.1% DMSO. The plates were incubated in a 5% CO_2_ incubator (Shanghai Lishen Scientific Equipment Co. Ltd., Shanghai, China) for 24, 48, or 72 h at 37 ± 1°C after being treated with the extracts and standards. MTT assay was performed to determine the cell viability level of each treatment.

### Determination of Cell Viability by MTT Assay

Cell viabilities of HepG2, RIN-5F, and 3T3-L1 cells were determined according to the procedure described by Khoo et al. with some modifications (24). After removing the medium, the plate was washed with cold PBS twice and added with 50 μL of DMEM medium containing 0.5 mg/mL thiazolyl blue tetrazolium bromide (MTT). The MTT was dissolved in PBS. After the cells were incubated in the CO_2_ incubator for 4 h, the medium was removed, and 150 μL of dimethyl sulfoxide (DMSO, cell culture grade) was added to dissolve the formazan crystal formed inside the cells. After 20 min incubation in the CO_2_ incubator, the absorbance of formazan solution was measured by the microplate spectrophotometer at 570 nm. The control samples were prepared without adding the extract solutions inside the wells, where they demonstrated 100% cell viability.

### Hydrogen Peroxide-Induced Oxidative Stress

The determination of hydrogen peroxide (H_2_O_2_)-induced oxidative stress was performed according to the method described by Coyle et al. (25). In brief, 20 μL of red pitaya extract or pure standard and 100 μL of the complete medium were added to each well of the 96-well plate containing 3T3-L1 cells (1.5 × 10^6^ cells/well) before the addition of 80 μL of 60 μM H_2_O_2_. The concentrations of the fruit extracts and standards ranged from 0.0625 to 1.0 mg/mL and 0.5 to 8.0 μg/mL, respectively. The plates were incubated in the CO_2_ incubator for 24 and 48 h. The cell viabilities were measured by MTT assay; antioxidant activities and EC_50_ values of the fruit extracts and standards were calculated. The total antioxidant status of the 3T3-L1 cells induced with H_2_O_2_ was determined using a FRAP-based test kit. The assay was performed according to the instructions provided with the test kit (Solarbio Life Sciences, Beijing, China).

### Cell Differentiation and Oil Red O Staining

The cell differentiation was induced by adapting the procedure reported by Sahib et al. (26). The 3T3-L1 cell differentiation was performed using the differentiation medium containing 10% FBS and a high-glucose DMEM mixture. The DMEM mixture contained 0.5 mM isobutylmethyl xanthine, 1 μM dexamethasone, and 1 μg/mL insulin in the DMEM. After adding 100 μL of the differentiation medium, the plate was incubated in the CO_2_ incubator for 72 h. The treatment groups were added with 50 μL of the fruit extracts (0.0625-1.0 mg/mL) or pure standards (0.5-8.0 μg/mL), whereas the control group was added with 50 μL of blank solution with the extract. The old medium was removed, washed three times with PBS, and replaced with a 100 μL of DMEM solution containing insulin (10 μg/mL) for another 72 h. The wells were replaced with 100 μL of fresh DMEM solution without adding insulin. The cells were fully differentiated on day 10. The lipid-lowering effect of the fruit extracts was determined by oil red O staining (ORO). The ORO staining kit was purchased from Solarbio Life Sciences (Beijing, China).

The ORO staining microplate assay was performed according to the instructions provided with the ORO staining kit and also referred to the method described in the literature (27) with some modifications. After the differentiated 3T3-L1 cells were washed twice, the cells were fixed with 100 μL of ORO fixative solution for 30 min. The fixative solution was removed, washed with distilled water (DW) twice, and soaked in 100 μL of 60% isopropanol for 5 min. After removing the isopropanol, 100 μL of ORO staining solution was added to the wells. The cells were stained with ORO stain for 20 min and washed with DW four times. The cells were also counterstained with 100 μL of Mayer’s hematoxylin solution for 2 min and washed with DW four times. ORO buffer (100 μL) was finally added into the wells, soaked for 1 min, and discarded. A 100 μL of isopropanol was added to the wells and incubated for 10 min in the CO_2_ incubator. The alcohol was used to dissolve the lipid accumulated. The absorbance was measured using the microplate spectrophotometer at 520 nm.

### Cell Cytotoxicity and Anticancer Potential

Cell cytotoxicity of the red pitaya pulp and peel extracts was evaluated based on the cell viability levels, where the cytotoxic effect of the sample or standard was tested using RIN-5F and 3T3-L1 cells. The cell viability of each treatment was determined using the MTT assay. The anticancer potential of the fruit samples and pure compounds was also determined by the cell culture method, where HepG2 cells were the typical cancerous cell line used.

### Statistical Analysis

All data were presented as mean ± standard error of the mean of three replicates. SPSS for Windows version 16.0 (SPSS Inc., Chicago, IL, United States) was used for data analysis. One-way analysis of variance (ANOVA) coupled with the least significant difference (LSD) test was used to determine the statistical differences between the two treatment groups. The significant differences were tested at a significance level of 0.05.

## Results and Discussion

### TBC, Betanin Content, TAC, and DPPH Radical Scavenging Activity

The TBC of the red pitaya pulp, peel, and colorant were estimated as 30.15 ± 0.03, 35.12 ± 0.01, and 18.35 ± 0.02 mg/g sample, respectively. The betanin content of these samples was calculated using equation 1. The methanol-extracted red pitaya peel had the highest TBC, but the TBC of the red pitaya pulp was significantly lower than the peel (*p* < 0.05). The TBC of the colorant was also lower than the methanol extracts. The result showed that red pitaya peel contained a high concentration of betacyanins. Huang et al. (28) also reported that red pitaya peel had higher total betacyanins than the pulp.

The result showed that the red pitaya pulp, peel, and colorant had betanin content of 7.44 ± 0.01, 9.44 ± 0.01, and 4.49 ± 0.01 mg/g sample, respectively. The betanin content of the methanolic extracts was about 25% of the TBC. The total anthocyanins estimated in the fruit pulp, peel, and colorant were 15.16 ± 0.02, 19.21 ± 0.01, and 9.15 ± 0.01 mg/g sample, respectively. Hence, the TAC in these samples was estimated to be about half the amount of the total betacyanins. The ANOVA results showed significant differences in these components among the fruit samples (*p* < 0.05).

**FIGURE 1:**
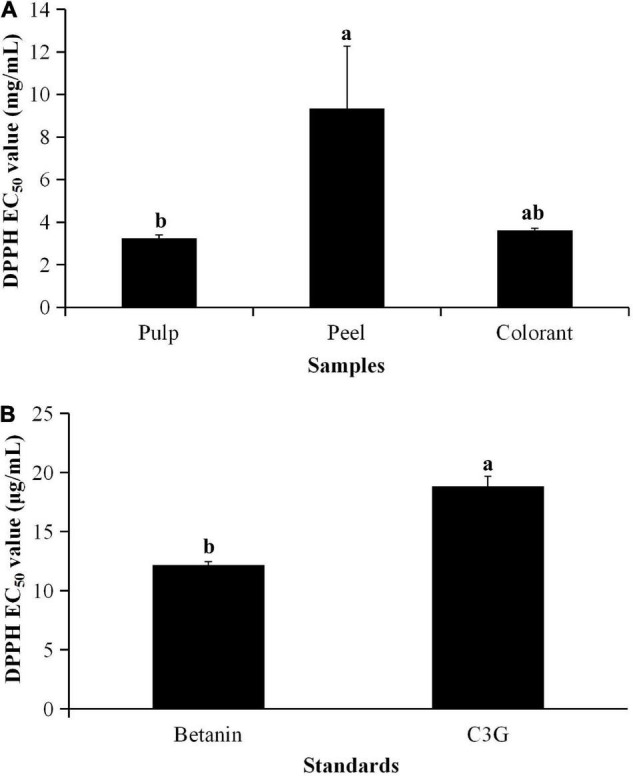
EC50 values of **(A)** pitaya samples and **(B)** standards calculated from DPPH radical scavenging activity. ^a^Different lowercase letters indicate significant differences between two samples at *p* < 0.05.

The result showed that the methanolic extract of the red pitaya peel had the highest EC_50_ value (Figure 1A), which was assessed based on the DPPH radical scavenging assay. The other samples had lower EC_50_ values (*p* < 0.05). The results indicated that the peel had a lower DPPH free radical scavenging activity, while the pulp had a higher activity. Also, the DPPH free radical scavenging activity in the red pitaya samples was lower than the pure compounds (Figure 1B) by 1,000 times because the pitaya samples were the crude extract. The fruit extracts might also contain organic acids, alkaloids, polysaccharides, or polypeptides.

Betanin is one of the betacyanin pigments (29). It is the main component determined in red pitaya. Although betanin and anthocyanin are red-colored pigments, anthocyanin could not occur together with betanin in the pitaya (30). Although the fruit contains anthocyanin, the anthocyanin content of most varieties of red pitaya is low. Literature also reported that betacyanins are the main bioactive detected in red pitaya (6). Moreover, betanin was used as the standard to quantify the TBC.

One study reported that the TBC of a fully ripened red pitaya pulp was as high as 8.72 mg/mL (5), whereas another study showed that red pitaya peel had a TBC of 7.72 mg/100 g of fresh weight (31). The TBC in Taiwanese red pitaya pulp and peel was 10.3 ± 0.22 and 13.8 ± 0.85 mg/100 g of fresh weight, respectively (4). The low amount of betacyanins determined in the red pitaya pulp extract could be due to the high percentages of sugar found in the pulp extract. In this study, the estimated sugar content of the crude pulp extract was 56% (Brix value of 2.7).

Literature showed that the total sugar content of red pitaya pulp (49.02 mg/100 g edible portion, EP) was higher than white pitaya (36.18 mg/100 g EP) and papaya (18.08 mg/100 g EP), whereas red pitaya peel had total sugar content of 7.66 mg/100 g EP (32). Another study reported that the glucose and fructose content of different varieties of red pitaya pulp ranged between 1.24 and 2.15% (33). The high amount of sugar in the crude pulp extract could attenuate the antioxidant ability of the extract. The high DPPH radical scavenging activity determined in the pitaya pulp extract could also be due to the ascorbic acid content in the pulp extract being higher than that of the peel extract. A previous study reported that ascorbic acid content in red pitaya pulp was 32.65 ± 1.59 mg/100 g of fresh pulp, but the ascorbic acid content in the whole fruit (18.94 ± 2.51 mg/100 g) was significantly lower than the fresh pulp (34).

Betanin is a better antioxidant than most anthocyanins and many other polyphenolic compounds (35, 36). The chemical structure of naturally occurring betanin has more hydroxyl groups than many polyphenolic compounds. Betanin contains three carboxyl groups, whereas this functional group does not exist in C3G. A review of anthocyanin also showed that a natural compound with a double bond conjugated to the keto group is a better antioxidant (16). Therefore, the antioxidant activity of betanin is higher than anthocyanin. Future studies need to determine the structure-activity relationship of the metabolites found in red pitaya pulp and peel.

### Total Antioxidant Status and Cell Viability of H_2_O_2_-Induced Oxidative Stress on 3T3-L1 Cell Line

**FIGURE 2:**
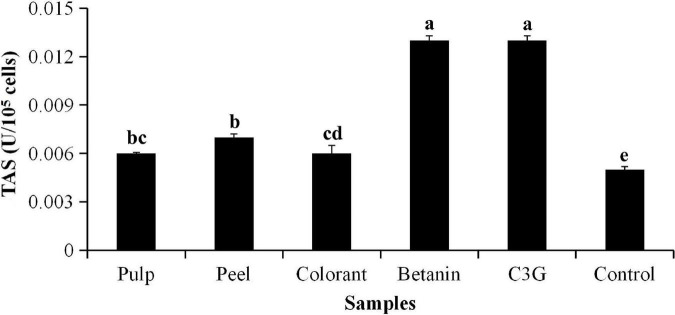
Total antioxidant status (TAS) of H_2_O_2_-induced oxidative stress to 3T3-L1 cells. ^a^TAS of 3T3-L1 cells induced by H_2_O_2_ for 24 h. ^b^Different lowercase letters indicate significant differences between two samples at *p* < 0.05.

**FIGURE 3:**
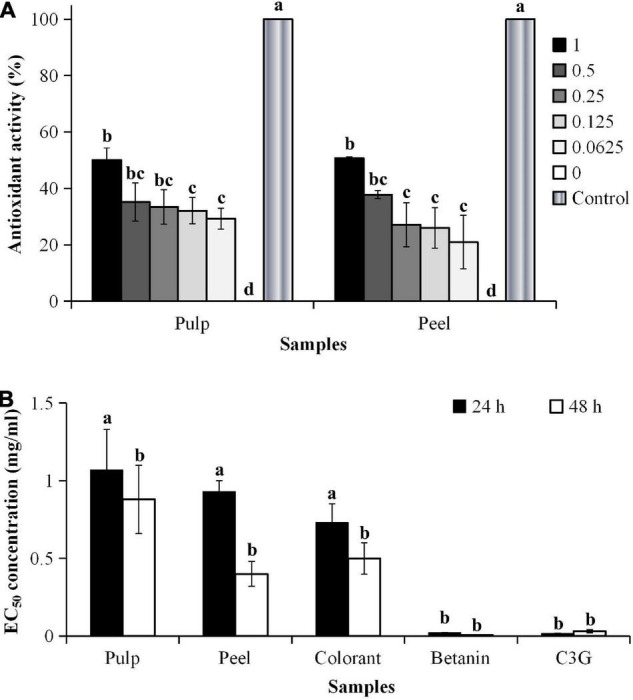
**(A)** Antioxidant activity and **(B)** EC_50_ values of H_2_O_2_-induced oxidative stress of 3T3-L1 cells. ^a^Calculated based on the non-H_2_O_2_-induced control, where the oxidative stress was induced for 48 h. ^b^IC_50_ values were calculated based on the cell viability of 3T3-L1 cells. ^c^Different lowercase letters indicate significant differences at *p* < 0.05.

The total antioxidant status (TAS) of the red pitaya extracts on inhibition of H_2_O_2_-induced oxidation is shown in Figure 2. The result showed that the TAS of the red pitaya peel extract was not significantly higher than the pulp extract (*p* > 0.05). The TAS of the pure standards (betanin and C3G) was significantly higher than that of the red pitaya samples (*p* < 0.01). An increase in the extract concentration of the red pitaya samples did not show increased antioxidant activity (Figure 3A). The antioxidant activities of the red pitaya samples with the highest extract concentration were significantly higher than the fruit samples with the lowest extract concentration (*p* < 0.05). Although the red pitaya peel extract had higher total betacyanins and a lower DPPH radical scavenging activity than the pulp extract, its TAS and antioxidant activity were similar to the pulp extract. Therefore, both pulp and peel had an equal inhibition effect against H_2_O_2_-induced oxidation.

The addition of hydrogen peroxide (H_2_O_2_) to the culture medium accelerated cell wall oxidation. The result showed that no significant differences were found in the inhibitory effect between the EC_50_ values of the pitaya samples (*p* < 0.05), except for the pure standards (Figure 3B). The results also showed that the cells treated with the pitaya extracts and colorant for 48 h had a better effect on inhibiting oxidative stress than the 24-h treatment.

**FIGURE 4:**
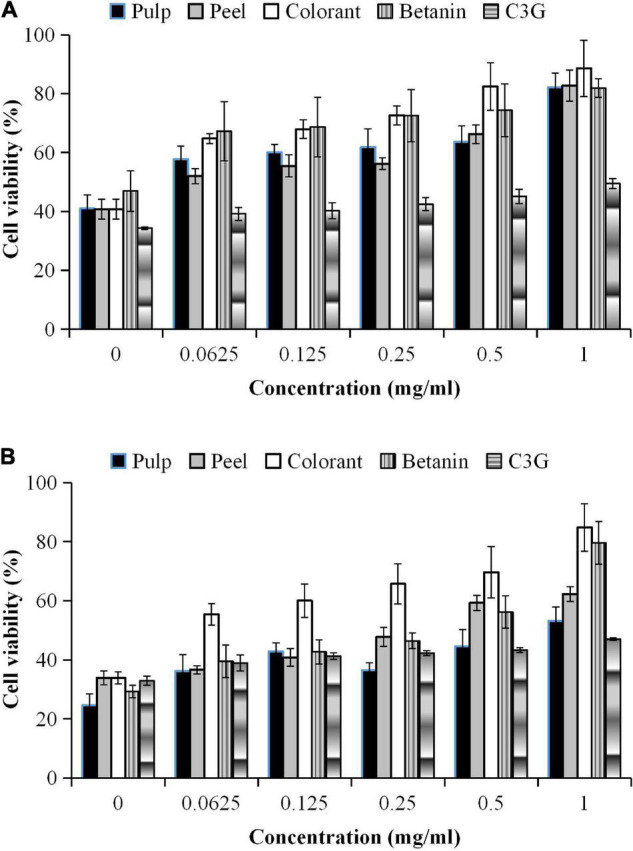
Percentages of cell viability of 3T3-L1 cells induced by H_2_O_2_ for **(A)** 24 h and **(B)** 48 h.

The inhibitory effect of the fruit samples was concentration-dependent (Figure 4). The oxidative stress-induced 3T3-L1 cells had higher cell viability, although the cells were treated with a high extract concentration. The cell viability was lower during the prolonged oxidative stress condition (48 h) than the shorter treatment duration (24 h). The high H_2_O_2_ level (60 μM) in the culture media accelerated the cell apoptosis rate during the prolonged treatment. The results showed that the oxidative stress-induced 3T3-L1 cells treated with the red pitaya extracts for up to 48 h had reduced cell viability. Also, the colorant and betanin were effectively inhibited the H_2_O_2_-induced oxidative stress during the 48-h treatment (37).

In this study, a higher extract concentration of the red pitaya showed a better inhibition effect. It might reduce the cell apoptosis rate. A 1 mg/mL betanin and betanin-rich samples had a better inhibitory effect than the other concentrations of the samples (Figure 4). At 48 h of treatment, the red pitaya peel extract-treated cells had up to 60% cell viability, but betanin-treated showed higher cell viability (∼80%). The peel extract showed better efficacy in inhibiting H_2_O_2_-induced oxidative stress than the pulp extract. The higher inhibitory effect of the red pitaya peel extract could be due to the higher betacyanins and anthocyanins content.

### *In vitro* Lipid-Reducing Effect

**FIGURE 5:**
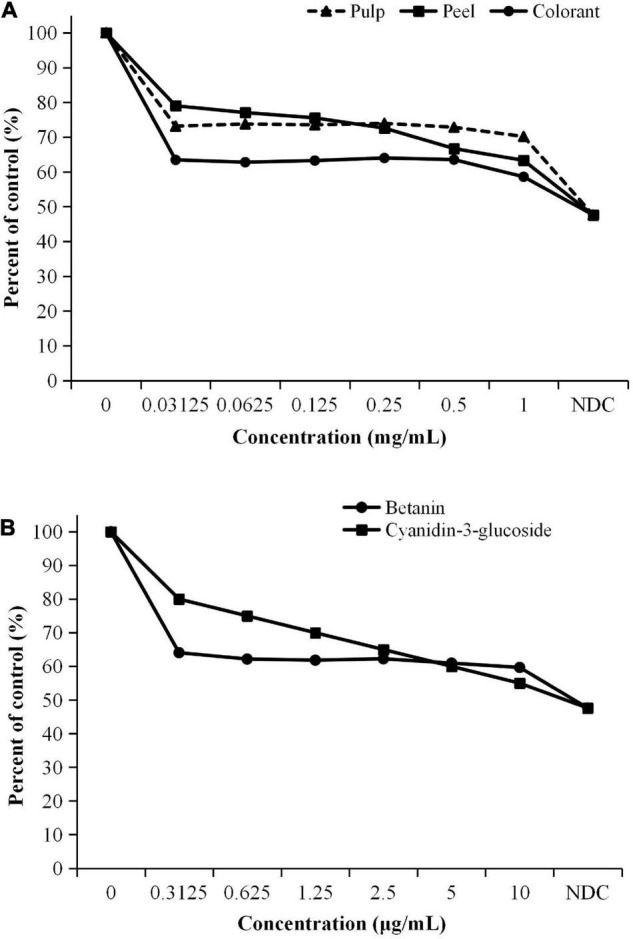
Inhibition effect of lipid accumulation in 3T3-L1 cells treated with **(A)** pitaya samples and **(B)** standards. ^a^Assessed based on the oil red O staining assay. ^b^NDC, non-differentiated cells.

The ORO staining was used to estimate lipid accumulation in the differentiated 3T3-L1 cells. As shown in Figure 5, the differentiated cells have a significantly higher intracellular fat content than the undifferentiated cells. The results showed that the inhibitory effect of the low concentrations (<0.25 mg/mL) of the fruit extracts was not as high as colorant (Figure 5). The methanolic extract of the red pitaya pulp at different sample concentrations did not significantly inhibit the lipid accumulation in the differentiated cells (*p* > 0.05). The peel extract had an increasing inhibitory trend but was not concentration-dependent. Nevertheless, C3G had a concentration-dependent inhibition of lipid accumulation. The differentiated cells treated with 1.0 mg/mL red pitaya pulp extract and colorant showed slight increase in the inhibitory rate at higher sample concentrations. The cells treated with 1 mg/mL colorant also had a higher inhibitory effect than the fruit extract-treated cells.

Based on the results obtained, the pitaya peel extract was more effective in inhibiting lipid accumulation in the differentiated 3T3-L1 cells at a higher sample concentration. A lower concentration of betanin was more effective than C3G in reducing lipid accumulation in the cells. On the contrary, a higher concentration of C3G (5 μg/mL) was needed to achieve an inhibition rate similar to betanin (Figure 5B). Red pitaya peel and colorant had the best inhibitory effect among the three samples. We can concluded that the treatment with the samples and pure compounds effectively inhibited lipid accumulation in the fully differentiated 3T3-L1 cells compared to the non-treated cells.

Cell differentiation increases the accumulation of lipids in the cells. Literature showed that betanin inhibited lipid accumulation in the differentiated 3T3-L1 cells through inhibiting peroxisome proliferator-activated receptor (PPAR) γ expression (17). The finding is further supported by the fact that the patients from both 16.2 and 35 mg betanin supplementation groups had improved lipid profiles, such as significant reductions in total triglycerides, total cholesterols, and low-density lipoprotein-cholesterol (38).

### MTT-Based Cytotoxic Effects

**FIGURE 6:**
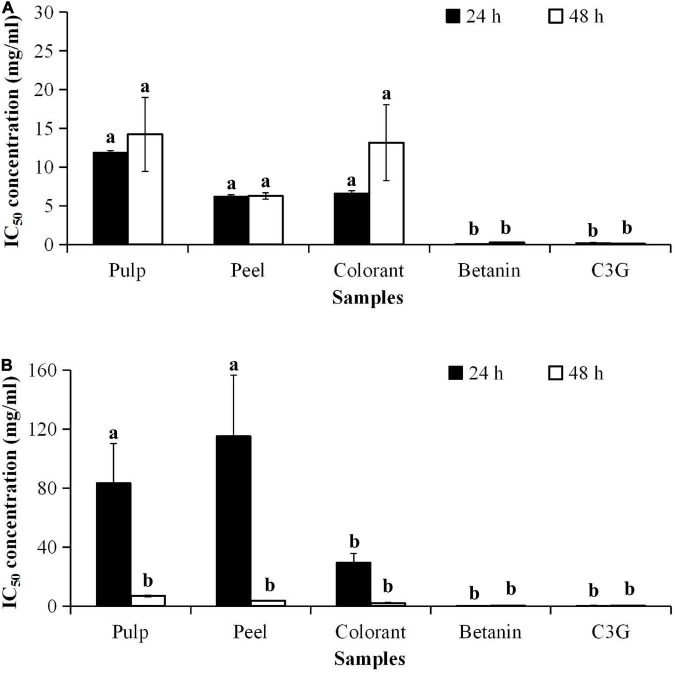
IC_50_ values of pitaya samples and standards for cytotoxicity of **(A)** 3T3-L1 and **(B)** RIN-5F cell lines treated for 24 and 72 h. ^a^Different lowercase letters indicate significant differences between different treatment times and between two samples at *p* < 0.05.

Cytotoxicity of the red pitaya extracts was determined using 3T3-L1 and RIN-5F cells. The anticancer effect of the fruit extracts was evaluated using the HepG2 cell line. The 3T3-L1 (Figure 6A) and RIN-5F cells (Figure 6B) treated with the fruit extracts did not have a notable cytotoxic effect. The results showed that the IC_50_ value of the peel extract-treated 3T3-L1 cells was as low as 6.21 mg/mL, while the IC_50_ value of the pulp extract was as high as 21.53 mg/mL. Lower IC_50_ values indicate higher cytotoxicity potential. The IC_50_ values of betanin and C3G-treated 3T3-L1 cells at 48 h were 0.24 and 0.15 mg/mL, respectively. Hence, only a high concentration of the extract or pure compound could induce apoptosis of mouse adipocytes. The cytotoxic effect of C3G was also 400 and 2 times higher than the fruit extract and betanin, respectively.

The treatment of the cultured cells with the methanolic extract of red pitaya pulp and peel for 24 h did not cause toxicity to the pancreatic islet cells (RIN-5F). The result showed that the commercially available colorant was more cytotoxic than the red pitaya extracts. Also, long-term treatment with the red pitaya extract or pure compound was somehow cytotoxic to the RIN-5F cells. Therefore, the short-term treatment with pulp and peel extracts of the red pitaya did not show any cytotoxicity to 3T3-L1 and RIN-5F cells.

Cell death is associated with the apoptosis of a cell. Natural substances either have low toxicity or not toxic to the normal cells. However, higher dosages of these substances might result in apoptotic cell death. It could be due to the increased oxidative stress in the cells. A previous study has shown that betanin is a non-toxic compound, and the IC_50_ value was 40 μmol/L (36). The IC_50_ value of betanin reported in the literature is closed to the value determined for the red pitaya pulp extract (39 μmol/L).

**FIGURE 7:**
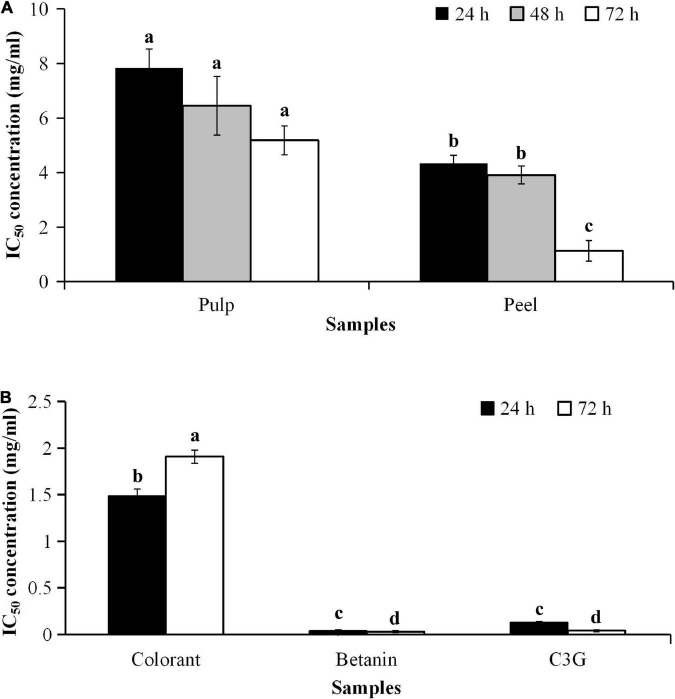
IC_50_ values of samples and standards for cytotoxicity of HepG2 cell line **(A)** treated with pulp and peel extracts for 24, 48, and 72 h, and **(B)** treated with colorant and standards for 24 and 72 h. ^a^Different lowercase letters indicate significant differences between different treatment times and between two samples at *p* < 0.05.

The cytotoxic effect of the bioactive compounds in the red pitaya extracts is associated with the anticancer potential. As betanin is not cytotoxic to the human body, its anticancer effect should be similar to the fruit extracts. The results showed that the IC_50_ values of the red pitaya extracts and colorant were higher than 1.0 mg/mL, but the IC_50_ value of betanin was lower (Figure 7). The high IC_50_ values denote a lower anticancer potential for all red pitaya samples studied. The result also showed that the red pitaya peel extract had higher anticancer potential than the pulp extract. It is because the IC_50_ value of the peel extract was significantly lower than the pulp extract (*p* < 0.05). Also, the anticancer potential of the colorant was higher than both red pitaya extracts. It could be because the colorant was more cytotoxic than the fruit extracts (Figure 6B).

The results showed that both betanin and C3G had low IC_50_ values. They had a similar anticancer potential, where the IC_50_ values of these compounds were 0.063 and 0.08 mM, respectively. These values were lower than 100 μM. As stated in a review paper, an effective anticancer drug must have an IC_50_ value lower than 10 μM; certain natural compounds with IC_50_ values as high as 100 μM had an inhibitory effect on H-Ras farnesylation in the mammalian cells (39). Therefore, both betanin and C3G had anticancer potential. Previous studies supported the findings of this study that betanin inhibited the growth of several cancer cell lines (11, 40), whereas C3G induced apoptosis in HS578T and Lewis lung carcinoma cells (41). As the IC_50_ value of the pitaya peel extract was higher than 100 μmol/L, it did not show anticancer potential. Pitaya peel extract is an alternative source of nutraceuticals for reducing oxidative stress due to the amounts of betacyanins and anthocyanins being higher than the pulp extract. The betanin-rich extracts might also indirectly reduce the risk of cancer.

The betanin-rich red pitaya is sweet and delicious; it is a functional food and harmless if consumed regularly. As betanin is less toxic than many other bioactive compounds, frequent consumption of red pitaya pulp with a high betanin content cannot cause food poisoning. Excessive intake of red pitaya is not advisable, especially the pitaya peel. Alkaloids and saponins are some of the main active compounds in the red pitaya peel on top of betanin. These compounds might cause food toxicity if taken orally in excessively high doses (42). Therefore, direct consumption of pitaya peel is not advisable. It is recommended to be used as an alternate source for betanin extraction.

## Conclusion

Red pitaya is beneficial to people who consume it regularly. The fruit has a high amount of red pigments, which are betacyanins. Betanin is one of the unique betacyanin pigments. In this study, the antioxidant activity of betanin was higher than anthocyanin, but both compounds had a comparable anticancer potential. The *in vitro* data revealed that the methanolic extracts of red pitaya, especially its peel extract, can reduce the risk of obesity as it had a lipid-reducing effect. Both pitaya extracts and colorant were not cytotoxic to 3T3-L1 and RIN-5F cells, and the anticancer potential of the fruit extracts was weak compared with betanin and C3G. In short, frequent consumption of red pitaya pulp may help to reduce oxidative stress and the risk of obesity. The pitaya peel extract is also a potent source of nutraceuticals for lowering the risk of metabolic-related diseases.

## Data Availability Statement

The original contributions presented in this study are included in the article/supplementary material, further inquiries can be directed to the corresponding authors.

## Author Contributions

HEK, XH, YZ, and JS conceptualized the work. HEK, ZL, YT, and JT performed all experiments. HEK, XH, and CL performed data analysis. HEK and YT wrote the manuscript. ZL, CL, YZ, and JS revised the manuscript. All authors contributed to the article and approved the submitted version.

## Conflict of Interest

The authors declare that the research was conducted in the absence of any commercial or financial relationships that could be construed as a potential conflict of interest.

## Publisher’s Note

All claims expressed in this article are solely those of the authors and do not necessarily represent those of their affiliated organizations, or those of the publisher, the editors and the reviewers. Any product that may be evaluated in this article, or claim that may be made by its manufacturer, is not guaranteed or endorsed by the publisher.
